# Kidney Replacement Therapies and Outcomes in Children With Crush Syndrome–Associated Kidney Injury

**DOI:** 10.1001/jamanetworkopen.2024.56793

**Published:** 2025-01-27

**Authors:** Demet Demirkol, Tolga Besci, Merve Havan, Dilek Karacanoğlu, Hasan Serdar Kıhtır, Dinçer Yıldızdaş, Muhterem Duyu, Abdulrahman Özel, Damla Pınar Yavaş Kocaoğlu, Naime Gökay, Fatih Durak, Merve Mısırlıoğlu, Mehmet Arda Kılınç, Şahin Sincar, Fatih Varol, Alper Köker, Tahir Dalkıran, Ayhan Yaman, Nihal Akçay, Sultan Göncü, Mey Talip, Emine Akkuzu, Hacer Uçmak, Tanıl Kendirli, Ülkem Barlas Koçoğlu, Erennur Tufan, Erdem Çebişli, Faruk Ekinci, Nurettin Onur Kutlu, Çelebi Kocaoğlu, Gülizar Koç, Mehmet Alakayav, Mustafa Çolak, Cihan Önder, Kübra Boydağ Güvenç, Nazan Ülgen Tekerek, Oğuz Dursun, Emrullah Aygüler, Ali Genco Gençay, Benan Bayrakçı

**Affiliations:** 1 Department of Pediatric Intensive Care, Istanbul Faculty of Medicine, Istanbul University, Istanbul, Turkey; 2 Department of Critical Care, Ankara University Faculty of Medicine, Istanbul, Turkey; 3 Department of Pediatric Intensive Care Medicine, Life Support Center, Hacettepe University, Ankara, Turkey; 4 Antalya Training and Research Hospital, University of Health Sciences, Antalya, Turkey; 5 Department of Pediatric Intensive Care, Çukurova University Faculty of Medicine, Adana, Turkey; 6 Pediatric Intensive Care Unit, Department of Pediatrics, Istanbul Medeniyet University, Göztepe Prof Dr Süleyman Yalcin City Hospital, Istanbul, Turkey; 7 Department of Pediatrics, Bağcılar Training and Research Hospital, University of Health Sciences, Istanbul, Turkey; 8 Department of Pediatric Intensive Care, Konya City Hospital, University of Health Sciences, Konya, Turkey; 9 Department of Pediatric Intensive and Critical Care, Adana Seyhan State Hospital, Adana, Turkey; 10 Department of Pediatric Intensive Care, İzmir Health Sciences University, Tepecik Training and Research Hospital, İzmir, Turkey; 11 Department of Pediatric Intensive Care, Faculty of Medicine, Mersin University, Mersin, Turkey; 12 Department of Paediatric Intensive Care, Basaksehir Cam and Sakura City Hospital, Istanbul, Turkey; 13 Pediatric Intensive Care Unit, Elazig Fethi Sekin City Hospital, Elazığ, Turkey; 14 Department of Pediatric Intensive Care, University of Health Science, Sancaktepe Şehit Profesör Dr. İlhan Varank Training and Research Hospital, Istanbul, Turkey; 15 Pediatric Intensive Care Unit, Akdeniz University School of Medicine, Antalya, Turkey; 16 Department of Pediatric Intensive Care, Necip Fazil City Hospital, Kahramanmaraş, Turkey; 17 Pediatric Intensive Care Unit, Department of Pediatrics, Istinye University, Istanbul, Turkey; 18 University of Health Sciences Turkey, Kanuni Sultan Suleyman Training and Research Hospital, Istanbul, Turkey; 19 Pediatric Intensive Care Unit, Ankara Training and Research Hospital, Ankara, Turkey; 20 Pediatric Intensive Care Unit, Prof Dr Cemil Tascioglu City Hospital, Istanbul, Turkey; 21 Department of Pediatric Intensive Care, Faculty of Medicine, Gazi University, Ankara, Turkey

## Abstract

**Importance:**

This study addresses the characteristics, kidney replacement therapy (KRT) modalities, and outcomes in children diagnosed with crush syndrome following an earthquake in Turkey.

**Objective:**

To analyze the associations of different KRT modalities with long-term dialysis dependency and length of stay (LOS) in the pediatric intensive care unit (PICU).

**Design, Setting, and Participants:**

This multicenter, prospective, and retrospective cohort study was conducted across 20 PICUs in Turkey. Participants included children diagnosed with crush syndrome after the 2023 Kahramanmaraş earthquake, and eligibility criteria included age, diagnosis, and need for KRT. Data were analyzed from August to October 2024.

**Exposure:**

Children diagnosed with crush syndrome who underwent KRT.

**Main Outcomes and Measures:**

The primary outcome was dialysis dependency at discharge. Secondary outcomes included LOS in the PICU.

**Results:**

The study included 183 pediatric patients (median [IQR] age, 158 (108-192) months; 49 [54.4%] males) with earthquake-related injury, of whom 90 required KRT. The median (IQR) time under the rubble was 25.7 (1-137) hours. At admission, 51 patients (56.6%) had stage 3 acute kidney injury, and the median (IQR) serum creatinine phosphokinase level was 15 555 (9386-59 274) IU/L. There was a significant association between the Kidney Disease—Improving Global Outcomes (KDIGO) stage at admission and serum creatinine phosphokinase level (area under the curve, 0.750; 95% CI, 0.621-0.879; *P* < .001). Among patients undergoing KRT, 33 (36.7%) received continuous venovenous hemodiafiltration, and 23 (25.6%) underwent intermittent hemodialysis (IHD). IHD treatment was the only independent factor associated with shorter PICU LOS (odds ratio [OR], 6.87; 95% CI, 1.54-30.67; *P* = .01). The dialysis dependency at discharge was higher in children who were transferred late to the PICU (β = 0.003; 95% CI, 0.001-0.005; *P* < .001) and those with a high Pediatric Trauma Score (β = 0.022; 95% CI, 0.003-0.041; *P* = 02). IHD was not statistically significantly associated with remaining dialysis-dependent at discharge (OR, 2.18; 95% CI, 0.53-8.98; *P* = .28). The overall mortality rate in the cohort was 6 patients (6.6%).

**Conclusions and Relevance:**

This cohort study found that children who were transferred late to intensive care and those with a high trauma score after earthquake-related crush injury were more likely to remain dialysis-dependent at discharge. Furthermore, KDIGO stage at admission was associated with elevated serum creatinine phosphokinase levels. These findings highlight the critical importance of early intervention and appropriate treatment in children with AKI following prolonged entrapment.

## Key Points

**Question:**

What kidney replacement therapy (KRT) modalities were used for children with crush syndrome–associated acute kidney injury (AKI) following a natural disaster and was KRT modality associated with dialysis dependency and other outcomes?

**Findings:**

In this cohort study of 90 pediatric patients with crush syndrome–associated AKI, intermittent hemodialysis was associated with shorter pediatric intensive care unit stay. There was no difference in dialysis-dependency in those treated with continuous venovenous hemodiafiltration vs intermittent hemodialysis.

**Meaning:**

Among pediatric patients with earthquake-related crush syndrome, KRT modality and was not associated with dialysis dependency at discharge.

## Teaser

This study addresses the characteristics, kidney replacement therapy (KRT) modalities, and outcomes in critically ill children diagnosed with crush syndrome following the 2023 Kahramanmaraș earthquake.

## Introduction

Millions of people worldwide experience natural disasters each year. At present, approximately 800 million people live in areas prone to earthquakes or at high risk of severe tropical hurricanes or typhoons.^1^ Serious injuries, like crush syndrome, frequently result from primary disasters, whether natural or human-induced.

Crush injury occurs with direct physical trauma to the body from an external force.^2,3,4,5,6,7^ During earthquakes, anywhere from 3% to 20% of mass casualties experience crush injury.^8,9^ Crush syndrome is the severe systemic manifestation of muscle and end-organ injury^5^ and is the second leading cause of death after destructive disasters. The injury’s cellular damage and muscle necrosis release substances such as myoglobin, potassium, phosphorus, and uric acid into the bloodstream.^2,6,10^ These substances can precipitate various complications, including acute kidney injury (AKI), hypotension, and acidemia. Among the subset of individuals who sustain crush injury, 40% to 70% develop crush syndrome.^2,3,4^ Compared with adults, there are limited data on crush syndrome in children. In a study conducted at a single center during the 1999 Marmara earthquake, acute kidney failure developed in 35% of 20 children diagnosed with crush syndrome.^11^

On February 6, 2023, 2 major earthquakes with magnitudes of 7.7 and 7.6 struck the districts of Pazarcık and Elbistan in Kahramanmaraş, Turkey, affecting 11 provinces and 110 000 km^2^, with a 350 km length.^12^ These earthquakes resulted in at least 50 500 deaths and more than 120 000 injuries. The children rescued from the earthquake were transported to hospitals in a different region of Turkey. As pediatric intensive care physicians in Turkey, we aimed to provide children with earthquake-related injury with the most effective treatment approaches. However, we observed that there are no treatment guidelines and very limited data for children diagnosed with crush syndrome. Therefore, following the Kahramanmaraş earthquake, we wanted to share our experiences regarding children undergoing kidney replacement therapy (KRT) in the pediatric intensive care unit (PICU). In this multicenter study, we summarize the characteristics, applied KRT methods, and outcomes of children diagnosed with crush syndrome and undergoing KRT.

## Methods

This cohort study was approved by the ethics committee of the Istanbul Faculty of Medicine. Strict adherence to patient data confidentiality and ethical standards in clinical research, per the Declaration of Helsinki, was maintained throughout the study. Informed consent was obtained from the parents or guardians of the participants in the study. This study follows the Strengthening the Reporting of Observational Studies in Epidemiology (STROBE) reporting guideline for cohort studies.

### Study Design and Setting

A study was conducted among children seriously injured by the 2023 Kahramanmaraş earthquake who were admitted to 20 different hospitals in Turkey. The study included patients aged between 1 month and 18 years who had crush syndrome–associated AKI and received KRT (eFigure 1 in Supplement 1). The study examined the clinical and laboratory variables and treatment modalities for crush syndrome–related AKI. Finally, we investigated the factors associated with the PICU length of stay (LOS) and dialysis dependency at discharge.

### Participants

A total of 90 children who had AKI and received KRT because of earthquake-related crush syndrome were included in the study. Patients dead on arrival, with inaccurate records, with prior chronic kidney disease, and who did not meet the diagnostic criteria for both crush syndrome and AKI were excluded from the study.

### Data Collection

Patient information and data were obtained through special questionnaires sent to the study centers. Responses to questionnaires were completed by the senior pediatric intensivist and 1 attending physician at each site.

The checklist used for data collection included demographic variables (age, sex, weight); history of underlying diseases; time under the rubble; type of injury (fracture, soft tissue injury); vital signs on admission to PICU (blood pressure, pulse rate, respiratory rate, level of consciousness); illness severity and organ dysfunction scores; laboratory findings (muscle enzymes, kidney function tests, electrolyte profile, and urinalysis); type, method, and duration of KRT applied; and outcome (fasciotomy, amputation, sepsis, multiple organ failure, acute respiratory failure, dialysis dependency, and death).

Some of the children had received KRT before being referred to the respective centers; however, it was only possible to obtain data on some treatments administered. The documented KRT was recorded. A senior pediatric intensivist and 1 attending physician were responsible for collecting data at each site. Considering the critical conditions at the time of the earthquake, some checklists were completed prospectively and at the time of the patient’s admission to the hospital and others were completed retrospectively using the patients’ medical records.

### Definitions

In this study, we applied several prespecified definitions for statistical analyses and for reporting the results. Mild rhabdomyolysis was defined as creatinine phosphokinase (CK) enzyme as serum level greater than 300 IU/L and less than 1000 IU/L on the first day of admission. Moderate and severe rhabdomyolysis (crush injury) was defined as CK enzyme serum level of at least 1000 IU/L on the first day of admission without any evidence of systemic complications. Crush syndrome was defined as CK enzyme serum level of at least 1000 IU/L on the first day of admission, along with the presence of systemic complications (AKI, sepsis, multiple organ failure, and acute respiratory failure). KRT was defined as the use of peritoneal dialysis, intermittent hemodialysis (IHD), or continuous KRT. AKI was diagnosed based on increased serum creatinine, decreased estimated glomerular filtration rate, or decreased urine output. The definition of oliguria at presentation was based on the report of decreased urine volume; however, subsequent oliguria was defined as urinary flow rate less than 0.5 mL/kg/h. The Kidney Disease–Improving Global Outcomes (KDIGO) definitions were used for grading AKI.^13^

### Statistical Analysis

This study used descriptive statistics, such as mean, SD, median, IQR, range, frequency, and ratio values. The distribution of variables was assessed using the Kolmogorov-Smirnov test. The Mann-Whitney *U* test was used to analyze quantitative independent variables. For the analysis of qualitative independent variables, the χ^2^ test was used, and when the conditions for the χ^2^ test were not met, Fisher exact test was used.

The receiver operating characteristic (ROC) curve was performed to evaluate factors associated with AKI severity and dialysis dependence at discharge. The area under the ROC (AUC) was calculated to assess the accuracy of these estimates, with higher AUC values indicating better discriminative ability. Optimal cutoff points were determined using the Youden index to balance sensitivity and specificity.

In cases where data were missing from the forms, the analysis was conducted using the complete data. The significance level was investigated using univariate and multivariate logistic regression. We used SPSS software version 27.0 (IBM). *P* values were 2-sided, and statistical significance was set at *P* < .05. Data were analyzed from August to October 2024.

## Results

A total of 183 pediatric patients (median [IQR] age, 158 (108-192) months; 49 [54.4%] males) with earthquake-related injury were admitted to 20 PICUs, 90 of whom required KRT (eFigure 1 in Supplement 1). The patients were transferred to PICUs within a median (range) of 46 (1-350) hours. Table 1 presents patient demographic characteristics, severity of injury findings, and interventions performed.

**Table 1.** Demographic Characteristics and Injury Severity Findings of Patients

Characteristic	Patients, No. (%)
Gender	
Male	49 (54.4)
Female	41 (45.6)
Age, median (IQR), mo	158 (108-192)
PRISM III score, median (IQR)	10 (6-14)
PELOD score, median (IQR)	10 (9-12)
Vasoactive-inotropic score, median (IQR)	10 (0.0-50.0)
Organ Failure Index, median (IQR)	2 (1-4.0)
Pediatric Trauma Score, median (IQR)	8 (5-10.0)
Time under the rubble, median (IQR), h	26 (1-137)
Body surface area injured, %	
<10	32 (35.6)
10-20	26 (28.9)
>20	32 (35.6)
Interventions^a^	
Fasciotomy	50 (55.6)
Amputation	32 (35.5)
Hyperbaric oxygen	45 (50.0)
Vasoactive infusion	32 (35.5)
Extracorporeal treatment modality	
IHD	23 (25.6)
CVVHD	13 (14.4)
CVVHDF	33 (36.7)
IHD and CKRT	19 (21.1)
SCUF	2 (2.2)

Abbreviations: CKRT, continuous kidney replacement therapy; CVVHD, continuous venovenous dialysis; CVVHDF, continuous venovenous hemodiafiltration; IHD, intermittent hemodialysis; PELOD, pediatric logistic organ dysfunction syndrome; PRISM III, pediatric risk of mortality score III; SCUF, slow continuous hemofiltration.

^a^ Any patient could receive more than 1 intervention, so the total percentage of interventions exceeds 100%.

Table 2 presents the patient characteristics and laboratory findings at admission stratified by KDIGO AKI stages. There were 12 patients (13.3%) in stage 1, 27 patients (30.0%) in stage 2, and 51 patients (56.7%) in stage 3. Notably, we observed a statistically significant difference in serum CK levels by KDIGO stage (median [IQR], 15 555 [9386-59 274] IU/L for stage 1 vs 66 996 [22 000-132 000] IU/L for stage 2; *P* = .01), indicating an association between higher CK levels and more advanced AKI stages. This suggests that CK levels serve as a potential biomarker for assessing AKI severity at the time of admission. ROC curve analyses for estimating patients with KDIGO stages 2 and 3 demonstrated that serum CK levels of 25 812 IU/L or higher before the initiation of KRT were significantly associated with severe AKI (AUC, 0.750 [95% CI, 0.621-0.879]; *P* < .001) (eFigure 2 in Supplement 1).

**Table 2.** KDIGO Stage and Patient Characteristics at Admission

Variable	KDIGO stage, median (IQR)			*P* value
	1 (n = 12)	2 (n = 27)	3 (n = 51)	
Pediatric Trauma Score	9 (8-9)	9 (5-12)	7 (4-9)	.19^a^
Organ Failure Index	2 (1-3)	2 (1-3)	3 (1-5)	.11^a^
Time under the rubble, h	14 (9-40)	12 (8-30)	20 (10-36)	.78^a^
Body surface area injured, No. (%)				
<10%	5 (41.7)	13 (48.1)	14 (27.5)	.18^b^
10%-20%	2 (16.7)	9 (33.3)	15 (29.4)	
>20%	5 (41.7)	5 (18.5)	22 (43.1)	
Biochemical values before KRT, median (IQR)				
Creatinine, mg/dL	0.87 (0.5-1.7)	3.2 (1.8-4.7)	3.7 (2.5-5.2)	<.001^a^
Calcium, mEq/L	7.3 (6.2-7.9)	7.1 (6.8-7.7)	7.0 (6.3-7.8)	.51^a^
Creatinine phosphokinase, IU/L	15 555 (9386-59 274)	66 996 (22 000-132 000)	60 000 (30 000-119 406)	.01^a^
Potassium, mEq/L	5.5 (4.7-6.6)	5.5 (4.6-6.5)	5.8 (5.1-6.6)	.74^a^
Phosphorus, mg/dL	6.3 (4.7-7.5)	6.6 (6-7.6)	7.2 (6-9)	.13^a^
Lactate, md/dL	22.07 (13.51-34.23)	27.03 (17.12-37.84)	17.12 (11.71-36.04)	.30^a^
BUN, mg/dL	28.2 (18.3-36)	47 (32.7-66.6)	61 (43.5-81.7)	<.001^a^
HCO_3_, mEq/L	17.3 (16-20.3)	18 (15.4-21.9)	17.1 (14.6-23)	.99^a^

Abbreviations: BUN, blood urea nitrogen; HCO_3_, bicarbonate; KDIGO, Kidney Disease—Improving Global Outcomes; KRT, kidney replacement therapy.

SI conversion factors: To convert creatinine to micromoles per liter, multiply by 76.25; potassium to millimoles per liter, multiply by 1; lactate to millimoles per liter, multiply by 0.111; BUN to millimoles per liter, multiply by 0.357; HCO_3_ to millimoles per liter, multiply by 1.

^a^ Calculated with Mann-Whitney *U* test.

^b^ Calculated with Pearson χ^2^ test.

The distribution of KRT modalities was as follows: 33 patients (36.7%) received continuous venovenous hemodiafiltration (CVVHDF), 23 patients (25.6%) received IHD, 19 patients (21.1%) received a combination of IHD and continuous KRT, 13 patients (14.4%) received continuous venovenous dialysis (CVVHD), and 2 patients (2.2%) received slow continuous ultrafiltration. Heparin was used as the anticoagulant method in 37 patients (59.7%). The median (range) PICU LOS was 14.5 (1-142) days. The overall mortality rate was 6 patients (6.6%).

### Factors Associated With PICU LOS

The median (IQR) LOS in the PICU was 14.5 (7-30) days, with 38 patients (44.7%) remaining in the PICU for more than 14 days. Table 3 outlines the key factors associated with prolonged PICU LOS (ie, >14 days). Patients in the prolonged LOS group had significantly higher Organ Failure Index and Vasoactive Index Scores than those with PICU LOS of 14 days or less. Additionally, the median time under rubble was notably longer in patients with prolonged LOS. The Pediatric Trauma Score was significantly lower in patients with prolonged LOS, indicating a higher severity of trauma.

**Table 3.** Association of Patient and Treatment Characteristics With Pediatric Intensive Care Unit Length of Stay

Variable	Pediatric intensive care unit length of stay		*P* value
	≤14 d (n = 41)	>14 d (n = 49)	
Age, median (IQR), mo	163 (116-193)	156 (96-192)	.42^a^
Male gender, No. (%)	18 (43.9)	31 (63.3)	.24^b^
Time under the rubble, median (IQR), h	12 (6-28)	20 (11-40)	.04^a^
PRISM III, median (IQR)	11 (7-12)	10 (6-17)	.40^a^
PELOD II score, median (IQR)	10 (10-11)	11 (7-12)	.18^a^
Organ Failure Index, median (IQR)	1 (1-3)	3 (1-4)	.04^a^
Pediatric Trauma Score, median (IQR)	9 (6-12)	6 (4-9)	.003^a^
Vasoactive-inotropic score, median (IQR)	0 (0-25)	20 (5-110)	.005^a^
Hyperbaric oxygen treatment, No. (%)			
Yes	17 (41.5)	28 (57.1)	.33^b^
No	24 (58.5)	21 (42.9)	
Fasciotomy, No. (%)			
Yes	19 (46.3)	31 (63.3)	.17^b^
No	22 (53.7)	18 (36.7)	
Amputation, No. (%)			
Yes	10 (24.4)	22 (44.9)	.03^b^
No	31 (75.6)	27 (55.1)	
Body surface area injured, No. (%)			
<10%	19 (46.3)	13 (26.5)	.28^b^
10%-20%	10 (24.4)	16 (32.7)	
>20%	12 (29.3)	20 (40.8)	
Pre-KRT KDIGO stage, No. (%)			
1	5 (12.2)	7 (14.3)	.09^b^
2	17 (41.5)	10 (20.4)	
3	19 (46.3)	32 (65.3)	
KRT modality, No. (%)			
CVVHDF	16 (39.0)	17 (34.7)	<.001^b^
IHD	18 (43.9)	5 (10.2)	
IHD and CKRT	5 (12.2)	14 (28.6)	
CVVHD	2 (4.9)	11 (22.4)	
SCUF	0 (0.0)	2 (4.1)	
CKRT vs IHD, No. (%)			
IHD	18 (43.9)	5 (10.2)	.006^b^
CKRT	23 (56.1)	44 (89.2)	

Abbreviations: CKRT, continuous kidney replacement therapy; CVVHD, continuous venovenous hemodialysis; CVVHDF, continuous venovenous hemodiafiltration; IHD, intermittent hemodialysis; KDIGO, Kidney Disease—Improving Global Outcomes; KRT, kidney replacement therapy; PELOD II, Pediatric Logistic Organ Dysfunction II; PRISM III, Pediatric Risk of Mortality Score III; SCUF, slow continuous ultrafiltration.

^a^ Calculated using Mann-Whitney *U* test.

^b^ Calculated using Pearson χ^2^ test.

The incidence of amputation was also higher in the group with prolonged PICU LOS. Although the pre-KRT KDIGO stage was numerically higher in patients with prolonged LOS, suggesting more severe kidney dysfunction, the difference was not statistically significant. The analysis also found that IHD was less frequently used in patients with prolonged LOS, whereas CVVHD was significantly more common in prolonged LOS.

Table 4 presents the logistic regression analysis identifying factors associated with LOS in the PICU. In the univariate analysis, the Pediatric Trauma Score was significantly associated with shorter LOS (β = −0.042; 95% CI, −0.069 to −0.016; *P* = .002), indicating a protective association for LOS. However, this association lost significance in the multivariate model (β = 0.863; 95% CI, 0.738 to 1.008; *P* = .06). Amputation showed an initial significant association with LOS (OR, 1.07; 95% CI, 1.17 to 7.22; *P* = .02), but this did not hold in the adjusted model. Importantly, IHD remained a robust risk factor, maintaining statistical significance in both univariate (OR, 6.57; 95% CI, 2.16 to 20.02; *P* < .001) and multivariate (OR, 6.87; 95% CI, 1.54 to 30.67; *P* = .01) analyses, highlighting its independent association with PICU LOS. Continuous KRT demonstrated a significant univariate association with PICU LOS (OR, 3.31; 95% CI, 1.61 to 6.78; *P* = .001) but did not retain significance in the multivariate model (Table 4).

**Table 4.** Regression Analysis of Factors Associated With PICU LOS and Dialysis Dependency at Discharge

Variable	Univariate regression model		Multivariate regression model	
	β (95% CI)	*P* value	β (95% CI)	*P* value
**PICU LOS**				
Linear regression				
Time under the rubble, h	0.004 (−0.001 to 0.008)	.09	NA	NA
Organ Failure Index	0.033 (−0.030 to 0.095)	.30	NA	NA
Pediatric Trauma Score	−0.042 (−0.069 to −0.016)	.002	0.863 (0.738 to 1.008)	.06
Vasoactive Inotropic Score	0.001 (−0.001 to 0.003)	.31	NA	NA
Logistic regression, OR (95% CI)				
Amputation	1.07 (1.17 to 7.22)	.02	1.12 (0.36 to 3.48)	.84
Intermittent Hemodialysis	6.57 (2.16 to 20.02)	<.001	6.87 (1.54 to 30.67)	.01
CKRT	3.31 (1.61 to 6.78)	.001	0.45 (0.13 to 1.56)	.21
**Dialysis dependency at discharge**				
Linear regression				
PICU transfer duration	0.003 (0.001 to 0.004)	<.001	0.003 (0.001 to 0.004)	<.001
Pediatric trauma score	0.025 (0.004 to 0.047)	.02	0.022 (0.003 to 0.041)	.03
Logistic regression, OR (95% CI)				
CKRT mode	1.15 (0.8 to 1.65)	.47	NA	NA
Intermittent hemodialysis	0.37 (0.12 to 1.16)	.09	NA	NA

Abbreviations: CKRT, continuous kidney replacement therapy; LOS, length of stay; NA, not applicable; OR, odds ratio; PICU, pediatric intensive care unit.

### Factors Associated With Dialysis Dependency at PICU Discharge

Table 5 highlights the factors associated with dialysis dependency at discharge from the PICU. The PICU transfer duration after hospital admission was significantly longer in the dialysis-dependent group than in the nondependent group (median [IQR] duration, 96 [48-98] hours vs 24 [2-48] hours; *P* < .001). The Pediatric Trauma Score was significantly higher in the dialysis-dependent group compared with the nondependent group (median [IQR] score, 11.5 [6.5-12] vs 8 [5-9]; *P* = .02), indicating more severe trauma in patients who remained dependent on dialysis at discharge.

**Table 5.** Association of Patient and Treatment Characteristics With Dialysis Dependency at Discharge

Variable	Not dialysis-dependent (n = 74)	Dialysis dependent (n = 16)	*P* value
Age, median (IQR), mo	156 (96-192)	176.5 (154.5-196.5)	.12^a^
Male gender, No. (%)	39 (52.7)	10 (62.5)	.33^b^
Time under the rubble, median (IQR), h	16 (10-35)	8 (6-36)	.12^a^
PICU transfer duration, median (IQR), h	24 (2-48)	96 (48-98)	<.001^a^
PRISM III, median (IQR)	9 (6-14)	11 (9.5-12.5)	.44^a^
PELOD II score, median (IQR)	10 (9-11)	10 (10-12)	.60^a^
Organ Failure Index, median (IQR)	2 (1-4)	1 (1-2.5)	.10^a^
Pediatric Trauma Score, median (IQR)	8 (5-9)	11.5 (6.5-12)	.02^a^
Vasoactive-inotropic score, median (IQR)	14 (0-47.5)	90 (0-190)	.45^a^
Hyperbaric oxygen treatment, No. (%)			
Yes	34 (45.9)	11 (68.8)	.10^b^
No	40 (54.1)	5 (31.3)	
Fasciotomy, No. (%)			
Yes	39 (52.7)	11 (68.8)	.24^b^
No	35 (47.3)	5 (31.3)	
Amputation, No. (%)			
Yes	27 (36.5)	5 (31.3)	.69^b^
No	47 (63.5)	11 (68.8)	
Body surface area injured, No. (%)			
<10%	23 (31.1)	9 (56.3)	.14^b^
10%-20%	22 (29.7)	4 (25.0)	
>20%	29 (39.2)	3 (18.8)	
Pre-KRT KDIGO stage, No. (%)			
1	12 (16.2)	0 (0)	.22^b^
2	21 (28.4)	6 (37.5)	
3	41 (55.4)	10 (62.5)	
KRT modality, No. (%)			
CVVHDF	32 (43.2)	1 (6.3)	.03^b^
IHD	16 (21.6)	7 (43.8)	
IHD and CKRT	13 (17.6)	6 (37.5)	
CVVHD	11 (14.9)	2 (12.5)	
SCUF	2 (2.7)	0	
CKRT vs IHD, No. (%)			
IHD	16 (21.6)	7 (43.8)	.01^b^
CKRT	58 (78.4)	9 (56.2)	

Abbreviations: CKRT, continuous kidney replacement therapy; CVVHD, continuous venovenous hemodialysis; CVVHDF, continuous venovenous hemodiafiltration; IHD, intermittent hemodialysis; KDIGO, Kidney Disease—Improving Global Outcomes; KRT, kidney replacement therapy; PELOD II, Pediatric Logistic Organ Dysfunction II; PICU, pediatric intensive care unit; PRISM III, Pediatric Risk of Mortality Score III; SCUF, slow continuous ultrafiltration.

^a^ Calculated using Mann-Whitney *U* test.

^b^ Calculated using Pearson χ^2^ test.

There were no statistically significant differences between the dialysis dependent and nondependent groups in KDIGO stage. Other factors, such as age, sex, time spent under rubble, Organ Failure Index, and Vasoactive Index Score, were not significantly associated with dialysis dependency. Patients who were treated with IHD were more likely to remain dialysis-dependent (7 of 23 patients [30.4%] with IHD vs 2 of 13 patients [15.4%] with CVVHD), while those receiving CVVHDF had a significantly lower dependency rate (1 of 33 patients [3.1%]; *P* = .03). This association between the modality of KRT and dialysis dependency was further supported by a significant difference in the overall use of continuous KRT and IHD (9 of 67 patients [13.4%] vs 7 of 23 patients [30.4%]; *P* = .01).

Table 4 presents the regression analysis identifying factors associated with dialysis dependency at discharge. In the univariate analysis, PICU transfer duration was significantly associated with dialysis dependency (β = 0.003; 95% CI, 0.001-0.004; *P* < .001), a finding that remained significant in the multivariate model (β = 0.003; 95% CI, 0.001-0.004; *P* < .001), indicating that longer transfer duration was associated with higher odds of dialysis dependency. The ROC analysis also showed that increased time from hospital admission to PICU transfer was significantly associated with higher odds of dialysis dependency at discharge (AUC, 0.818; 95% CI, 0.711-0.925; *P* < .001). Notably, patients transferred to the PICU after the 47th hour (cutoff point) demonstrated a significantly higher likelihood of being discharged with dialysis dependency (eFigure 3 in Supplement 1). The Pediatric Trauma Score also showed a significant association in both univariate (β = 0.025; 95% CI, 0.004-0.047; *P* = .02) and multivariate analyses (β = 0.022, 95% CI, 0.003-0.041; *P* = .03), underscoring its role as a contributing factor.

Other variables, such as continuous KRT mode and IHD treatment, did not demonstrate significant associations in this context. These results highlight the importance of PICU transfer duration and Pediatric Trauma Score as independent factors associated with dialysis dependency at discharge.

### Comparison Between IHD and Other KRT Modalities

To understand the reasons behind the higher rates of dialysis dependency despite shorter LOS in the group receiving IHD, we analyzed the characteristics of children treated exclusively with IHD (eTable in Supplement 1). Patients in the IHD group had significantly shorter times under the rubble and lower Pediatric Trauma Score and Organ Failure Index compared with those treated with alternative KRT methods. Notably, the amputation rate was significantly lower in the IHD group. The shorter time under the rubble, lower trauma severity, fewer organ failure dysfunctions, and lower amputation rates in the IHD group likely explain the shorter hospital LOS. Although the odds of dialysis dependency at discharge among patients treated with IHD were numerically higher, this was not statistically significant.

## Discussion

In this cohort study, we assessed pediatric patients with crush syndrome who required KRT following a natural disaster. The findings highlighted significant outcomes related to dialysis dependency. We found that the time to access adequate PICU support and the severity of trauma were significantly associated with the development of major adverse kidney events at discharge.

The observed rates of AKI and KRT dependency in this cohort align with findings from previous disaster studies. Notably, 49.2% of the children in this study admitted to the PICU required KRT. This rate reflects the severity of injuries sustained during the earthquake and underscores the critical need for adequate kidney support. In the Wenchuan earthquake, 41.6% of patients with crush syndrome developed AKI.^14^ In the 1999 Marmara earthquake, 19% of the population in the disaster area were younger than 10 years, but only 2% of patients with crush-related AKI were younger than 10 years.^15^ In a study by Öven et al^16^ following the Maraş earthquake, a similar incidence of crush syndrome was found, at 2.6%. Sever et al^15^ studied 5302 patients injured in the Marmara earthquake in Turkey and reported that AKI development frequency due to crush syndrome was approximately 12%, and 9% of patients needed dialysis support. In a study by Karakaya et al^17^ conducted during the Maras earthquake, KRT was administered in 42.8% of hospitalized children.^17^ Probable reasons that could explain the difference in the reported statistics regarding AKI due to crush syndrome could be differences in the demographic data of the injured patients, time of the earthquake, time under the rubble, type of treatment used for patients, delay in initiation of prophylactic treatments, and difference in volume and combination of fluids used for treating dehydration in patients.^18^ The high incidence of AKI in our study may be due to the inclusion of patients with severe earthquake-related injuries.

Various tools can be used to screen patients at high risk of long-term adverse outcomes. The most straightforward screening uses available factors, such as serum muscle enzyme levels, clinical examination, and time under the rubble. With a longer time of direct pressure on muscle, broader surface of muscles under rubble (such as the thigh or lower extremity), and higher level of enzymes, we should expect a higher risk of systemic complications due to rhabdomyolysis. Regarding time under the rubble and the risk of kidney failure, there are contradictory opinions. Some reports suggest that entrapment duration is essential,^19,20,21^ whereas others suggest that the crushed area and burial duration are not associated with the degree of kidney damage.^6,22^ Two single-center analyses focusing on children with crush injury related to the Marmara disaster found that their mean time under the rubble was 18 hours^11^ or even 35 hours,^23^ considerably longer than time under the rubble in the analysis of the general Marmara population (12 hours); however, the incidence of crush syndrome was lower than that of the general population. In a case series by Sever et al,^24^ there was an apparent discordant finding because time under the rubble was negatively associated with the indicators of kidney function, such as admission serum creatinine level, duration of oliguria, number of hemodialysis sessions, and hospital LOS.^24^ These findings suggest that children have less frequent and milder injuries.^15^ Our study found no associations of AKI stage with the time under the rubble, affected surface area, or Organ Failure Index. However, an association was observed between the CK level at admission and the severity of AKI. In their 2024 study, Karakaya et al^17^ found that a kidney score based on glomerular filtration rate, CK level, time under rubble, presence of amputation or fasciotomy, and urine volume were effective biomarkers associated with KRT indication in pediatric patients with crush syndrome.^17^

During rhabdomyolysis, muscle cells break down, releasing large amounts of myoglobin, which plays a crucial role in pathogenesis. In our study, CK levels higher than 25 812 IU/L were associated with severe AKI, which shows the importance of myoglobin in rhabdomyolysis-induced AKI. Consequently, alongside supportive care, endeavors have been made to target myoglobin removal. Currently, limited evidence supports the clinical efficacy of diverse extracorporeal therapies for myoglobin elimination. KRT, including continuous KRT or IHD, plays a vital role in the treatment of rhabdomyolysis-induced AKI.^25^ Each type of dialysis is associated with advantages and drawbacks. Among KRT modalities, IHD offers several benefits, such as a high clearance rate of uremic solutes, the possibility to dialyze without anticoagulation, and the ability to treat several patients per day at the same dialysis post in the unit. However, a study by Meng et al^26^ suggested that the available clinical evidence does not show the benefit of KRT modes in preventing rhabdomyolysis-induced AKI.^26^ An animal experiment was performed to explore the direct protective effect of CVVH on the kidneys in the early stage of rhabdomyolysis.^27^ In that 2013 study,^27^ the hind legs of dogs were intramuscularly injected with 50% hypertonic glycerol to establish rhabdomyolysis, and 2 hours after the injection, CVVH was performed for 8 hours. The study confirmed that at the cellular and molecular levels, CVVH treatment mitigated myoglobin-induced mitochondrial damage by inhibiting the mitochondrial apoptotic pathway and cell apoptosis; the treatment also delayed the occurrence of oliguria and protected kidney function during the early stage of rhabdomyolysis development.^27^ In a study by Zeng et al^28^ that evaluated 3 randomized clinical trials involving 101 patients, although continuous KRT was associated with improved serum creatinine, blood urea nitrogen, and potassium levels; reduced duration of the oliguria phase; and reduced LOS, no significant differences were found in mortality rates compared with conventional therapy. The included studies did not report on long-term outcomes or prevention of AKI.^28^ Two small randomized clinical trials indicated better kidney recovery for continuous KRT compared with IHD in specific patient subsets^29^ or without achieving statistical significance,^30^ mirroring the findings of a meta-analysis of randomized clinical trials,^31^ potentially attributed to the small sample sizes. Numerous observational studies^32,33,34,35,36^ and a meta-analysis encompassing these studies^37^ reported significantly lower KRT dependence in patients treated with continuous KRT than those treated with IHD. Lastly, a secondary analysis of the STARRT-AKI trial found a reduced 90-day KRT dependence in patients initiating continuous KRT compared with those starting with IHD.^38^ We hypothesized that the slower ultrafiltration, less KRT-induced hypotension, and continuous clearance with continuous KRT (vs IHD) would result in less KRT dependence in patients with crush syndrome. In our study, patients treated with CVVHDF showed improved kidney recovery rates.

Recent findings, such as a 2024 study by Fuhrman et al,^39^ suggest that early initiation of KRT in children diagnosed with AKI is associated with improved survival and reduced dialysis dependency.^39^ Our findings that longer PICU transfer duration, higher Pediatric Trauma Score, and greater CK levels (>25 000 IU/L at admission), should serve as an alert for the timing of KRT in children diagnosed with crush syndrome.

### Strengths and Limitations

Our study has several notable strengths. Our results represent a contemporary, multicenter cohort of pediatric patients with crush syndrome. To our knowledge, our study is the first multicenter study conducted on children diagnosed with crush syndrome and treated with KRT. Similarly, it is the first study to our knowledge to show the association between the KRT modality and dialysis dependency.

This study has several limitations that should be considered when interpreting the results. First, the retrospective design may introduce selection bias, as data were collected from a specific cohort of pediatric patients with earthquake-related injury treated in 20 PICUs. This limits the generalizability of the findings to other populations or settings. Second, the reliance on existing medical records may result in incomplete data, particularly concerning clinical parameters and interventions that need to be systematically documented. This could affect the accuracy of the associations observed between variables. Third, the study’s observational nature does not allow for the establishment of causality. While significant associations were identified, it is essential to recognize that other unmeasured confounding factors could influence the outcomes. Fourth, the relatively small sample size of patients requiring KRT may limit the statistical power of the analyses and increase the potential for Ttpe I errors in the findings related to dialysis dependency and PICU LOS. Fifth, variations in clinical practice among the 20 participating PICUs, including differences in treatment protocols for KRT, could introduce variability in outcomes that may not be fully accounted for in the analysis.

## Conclusions

The findings of this cohort study suggest that children diagnosed with crush syndrome should be promptly transferred to centers where appropriate and effective intensive care support can be provided. This rapid intervention was significantly associated with the effectiveness of KRT and long-term kidney health. In disaster settings, especially for pediatric patients, timely implementation of suitable KRT strategies may reduce the risk of dialysis dependency and support recovery. In these patients, continuous KRT may reduce the risk of dialysis dependency.

## Notes

Supplement 1. **eTable.** Association Between Various Factors and RRT Mode **eFigure 1.** Flowchart of Patient Selection **eFigure 2.** ROC Curve Analysis for CK Levels in Predicting KDIGO Stages 2 and 3 **eFigure 3.** ROC Curve Analysis of PICU Transport Duration Predicting Dialysis Dependency at Discharge

Supplement 2.
Data Sharing Statement
