# Supplementary material for: Kidney Replacement Therapies and Outcomes in Children With Crush Syndrome–Associated Kidney Injury
Source: JAMA Netw Open. 2025 Jan 27;8(1):e2456793. doi: 10.1001/jamanetworkopen.2024.56793 (PMC11774091; doi:10.1001/jamanetworkopen.2024.56793)
Supplement: Supplement 1. — eTable. Association Between Various Factors and RRT Mode eFigure 1. Flowchart of Patient Selection eFigure 2. ROC Curve Analysis for CK Levels in Predicting KDIGO Stages 2 and 3 eFigure 3. ROC Curve Analysis of PICU Transport Duration Predicting Dialysis Dependency at Discharge [file jamanetwopen-e2456793-s001.pdf]

Supplemental Online Content

Demirkol D, Besci T, Havan M, et al. Kidney replacement therapies and outcomes in children with crush syndrome–associated kidney ınjury. *JAMA Netw Open*. 2025;8(1):e2456793. doi:10.1001/jamanetworkopen.2024.56793

eTable. Association Between Various Factors and RRT Mode

eFigure 1. Flowchart of Patient Selection

eFigure 2. ROC Curve Analysis for CK Levels in Predicting KDIGO Stages 2 and 3

eFigure 3. ROC Curve Analysis of PICU Transport Duration Predicting Dialysis Dependency at Discharge

This supplemental material has been provided by the authors to give readers additional information about their work.

eTable 1. Association Between Various Factors and RRT Mode

Variable	IHD Treatment (n=23)	Other RRT Modes (n=65)	P value
**Age, months, median (IQR)**	173 (117-200)	155 (101-191)	0.07 ^{m}
**Gender, Male, % (n)**	47.8 (11)	56.9 (37)	0.45 $^{\chi^2}$
**TPR, hours, median (IQR)**	12 (6-16)	22 (10.5-44)	**0.001** ^{m}
**PRISM III, median (IQR)**	10 (3-11)	11 (7-16)	**0.038** ^{m}
**PELOD II score, median (IQR)**	10 (10-10)	11 (9-12)	0.085 ^{m}
**OFI, median (IQR)**	1 (1-2)	2 (1-4)	**0.030** ^{m}
**PTS, median (IQR)**	12 (8-12)	6 (4-9)	**<0.001** ^{m}
**VIS, median (IQR)**	0 (0-4)	20 (0-63)	0.083 ^{m}
**Hyperbaric oxygen treatment , % (n)**			0.90 $^{\chi^2}$
Yes	52.2 (12)	50.8 (33)	
No	47.8 (11)	49.2 (32)	
**Fasciotomy, % (n)**			0.79 $^{\chi^2}$
Yes	52.2 (12)	55.4 (36)	
No	47.8 (11)	44.6 (29)	
**Amputation, % (n)**			**0.007** $^{\chi^2}$
Yes	13.0 (3)	44.6 (29)	
No	87.0 (20)	55.4 (36)	
**The surface area of the injured body, % (n)**			**0.038** $^{\chi^2}$
<10%	52.2 (12)	30.8 (20)	
10-20%	8.7 (2)	35.4 (23)	
>20%	39.1 (9)	33.8 (22)	
**Pre-RRT KDIGO stage, % (n)**			0.58 $^{\chi^2}$
I	13.0 (3)	13.8 (9)	
II	39.1 (9)	27.7 (18)	
III	47.8 (11)	58.5 (38)	

© 2025 Demirkol D et al. *JAMA Network Open*.

**RRT modality, % (n)**			**<0.001** $^{\chi^2}$
CVVHDF	0.0 (0)	50.8 (33)	
IHD	100.0 (23)	0.0 (0)	
IHD and CRRT	0.0 (0)	29.2 (19)	
CVVHD	0.0 (0)	20.0 (13)	

m: *Mann-Whitney U test,* ***χ^2:*** *Pearson chi-square test, significant p-values are shown in bold.*

Abbreviations*: PICU, Pediatric Intensive Care Unit; LOS, Length of Stay; IQR, Interquartile Range; TPR; time under the rubble; PRISM III, pediatric risk of mortality score III; PELOD II, pediatric logistic organ dysfunction II; OFI, organ failure index; PTS, pediatric trauma score; VIS, vasoactive index score; RRT, Renal Replacement Therapy; KDIGO, Kidney Disease: Improving Global Outcomes; CVVHDF, continuous venovenous hemodiafiltration; IHD, intermittent hemodialysis; CRRT, continuous renal replacement therapy; CVVHD, continuous venovenous hemodialysis; SCUF, slow continuous ultrafiltration.*

© 2025 Demirkol D et al. *JAMA Network Open*.

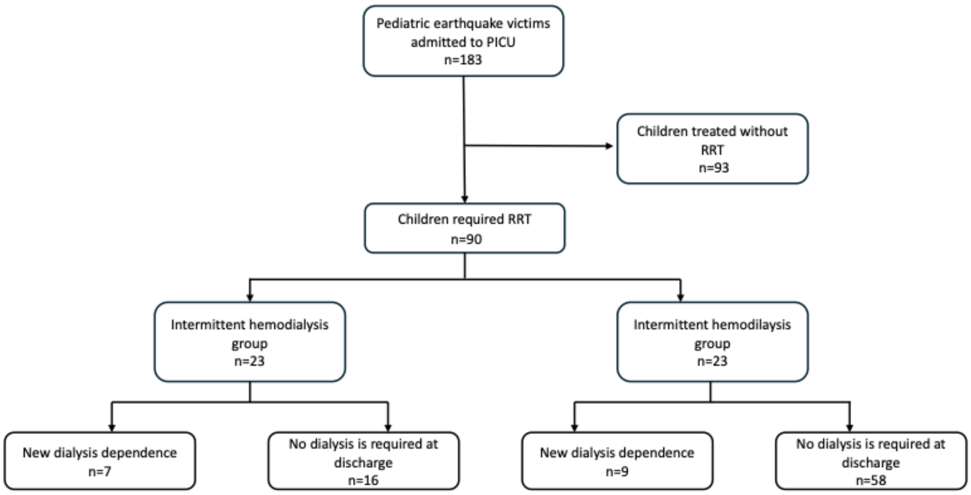

eFigure 1. Flowchart of patient selection

© 2025 Demirkol D et al. *JAMA Network Open*.

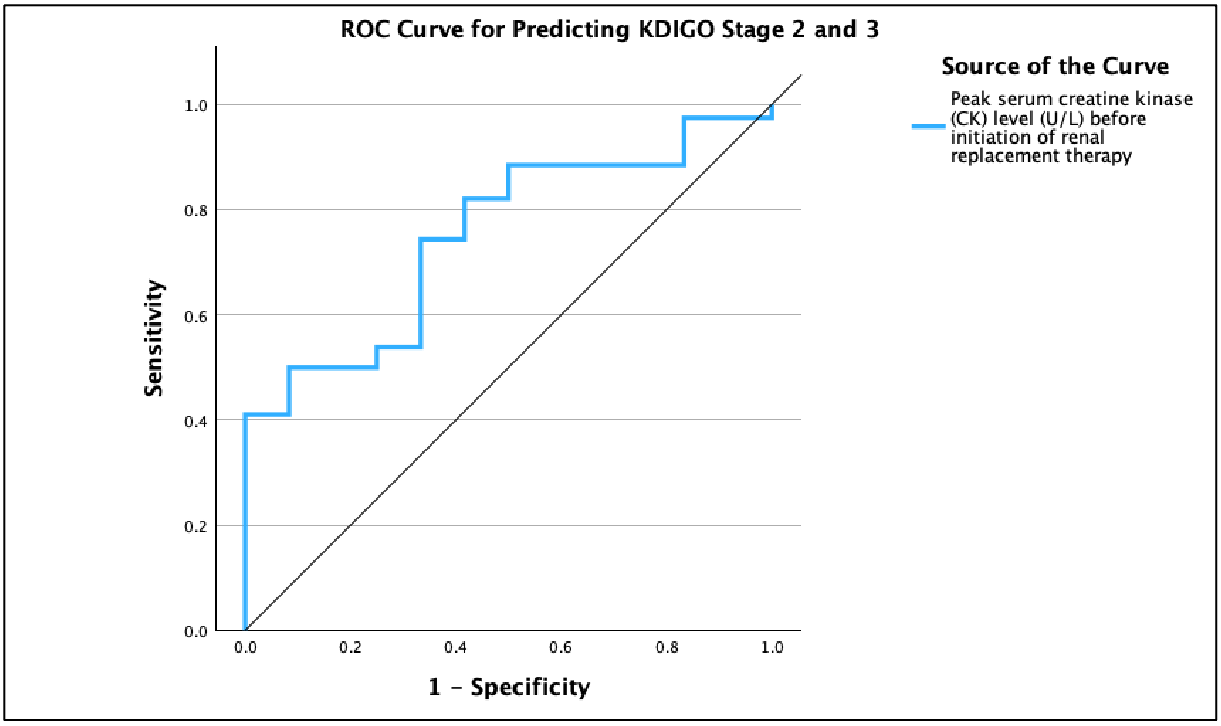

eFigure 2. ROC curve analysis for CK levels in predicting KDIGO stages II and III

© 2025 Demirkol D et al. *JAMA Network Open*.

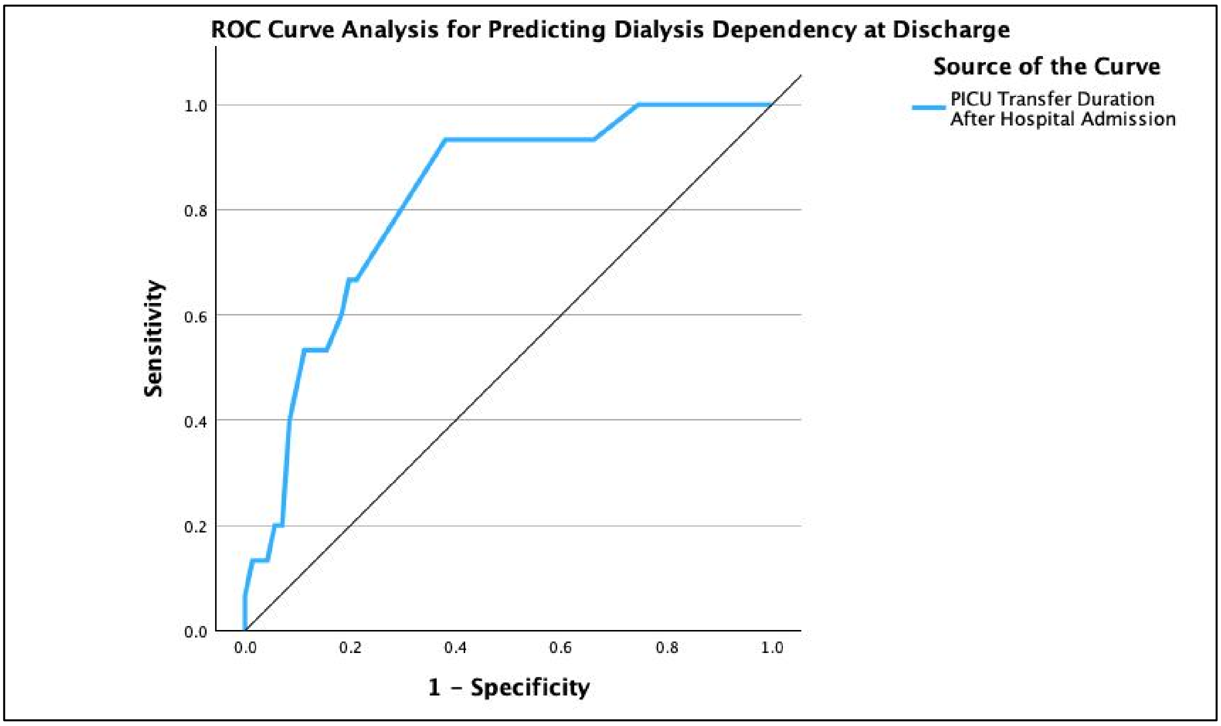

eFigure 3. ROC curve analysis of PICU transport duration predicting dialysis dependency at discharge

© 2025 Demirkol D et al. *JAMA Network Open*.
