# Supplementary material for: Kidney Replacement Therapies and Outcomes in Children With Crush Syndrome–Associated Kidney Injury
Source: JAMA Netw Open. 2025 Jan 27;8(1):e2456793. doi: 10.1001/jamanetworkopen.2024.56793 (PMC11774091; doi:10.1001/jamanetworkopen.2024.56793)
Supplement: Supplement 2. — Data Sharing Statement [file jamanetwopen-e2456793-s002.pdf]

Data Sharing Statement

Demirkol. Kidney Replacement Therapies and Outcomes in Children With Crush Syndrome–Associated Kidney Injury. *JAMA Netw Open*. Published January 27, 2025.
doi:10.1001/jamanetworkopen.2024.56793

Data
Data available: Yes
Data types: Deidentified participant data
How to access data: ddemirkol@istanbul.edu.tr
When available: With publication

Supporting Documents
Document types: Statistical/analytic code
How to access documents: ddemirkol@istanbul.edu.tr
When available: With publication

Additional Information
Who can access the data: Anyone requesting the data
Types of analyses: For any purpose
Mechanisms of data availability: With investigator support
Any additional restrictions: None
